# Effects of *Nosema ceranae* (Dissociodihaplophasida: Nosematidae) and Flupyradifurone on Olfactory Learning in Honey Bees, *Apis mellifera* (Hymenoptera: Apidae)

**DOI:** 10.1093/jisesa/ieaa130

**Published:** 2020-11-24

**Authors:** Heather Christine Bell, Corina N Montgomery, Jaime E Benavides, James C Nieh

**Affiliations:** Division of Biological Sciences, Section of Ecology, Behavior, and Evolution, University of California San Diego, Gilman Dr, La Jolla, CA

**Keywords:** *Nosema ceranae*, honey bee, olfactory conditioning, field-realistic, pesticide

## Abstract

The health of insect pollinators, particularly the honey bee, *Apis mellifera* (Linnaeus, 1758), is a major concern for agriculture and ecosystem health. In response to mounting evidence supporting the detrimental effects of neonicotinoid pesticides on pollinators, a novel ‘bee safe’ butenolide compound, flupyradifurone (FPF) has been registered for use in agricultural use. Although FPF is not a neonicotinoid, like neonicotinoids, it is an excitotoxic nicotinic acetylcholine receptor agonist. In addition, *A. mellifera* faces threats from pathogens, such as the microsporidian endoparasite, *Nosema ceranae* (Fries et al. 1996). We therefore sought 1) to increase our understanding of the potential effects of FPF on honey bees by focusing on a crucial behavior, the ability to learn and remember an odor associated with a food reward, and 2) to test for a potential synergistic effect on such learning by exposure to FPF and infection with *N. ceranae.* We found little evidence that FPF significantly alters learning and memory at short-term field-realistic doses. However, at high doses and at chronic, field-realistic exposure, FPF did reduce learning and memory in an olfactory conditioning task. Infection with *N. ceranae* also reduced learning, but there was no synergy (no significant interaction) between *N. ceranae* and exposure to FPF. These results suggest the importance of continued studies on the chronic effects of FPF.

Several governments, including members of the European Union and some provincial Canadian governments, have responded to concerns about impacts of neonicotinoid pesticides on nontarget taxa by restricting its use (European Commission 2018, Government of Ontario 2018). To counter these restrictions, major chemical manufacturers have begun to investigate the efficacy of other pesticides that are claimed to be potentially ‘bee-safe’. Bayer recently received approval flupyradifurone (FPF) for a wide range of agricultural applications. FPF was first commercially available in Honduras and Guatemala in 2014 (Nauen et al. 2015), and has since become available for use on a wide range of crops in the United States and elsewhere (Bayer registers first honeybee-low-toxic insecticide Flupyradifurone in China 2016; European Food Safety Authority [EFSA] 2016; U.S. Environmental Protection Agency 2015).

Flupyradifurone is a systemic pesticide based on a modification of the molecular structure of the phyto-derived compound, stemofoline (Nauen et al. 2015). Although FPF is classified as a butenolide, not a neonicotinoid, it is also a nAChR agonist, like the neonicotinoids (Jeschke et al. 2013, Nauen et al. 2015, Ihara et al. 2017). FPF has a binding affinity for nAChRs similar to that of neonicotinoids, and was designed explicitly to target Hemipterans, with the intention that these animals would be exposed to much greater doses of FPF via constant throughput of plant fluids than pollinators (Nauen et al. 2015).

The majority of honey bee pesticide studies focus on lethality and overall colony effects of pesticides (Johnson 2016). However, the effects of pesticides should be assessed on multiple aspects of pollinator health and biology (Decourtye et al. 2013). Olfactory learning is key to colony food intake and the ecosystem services provided by honey bees because it allows bees to associate odors with nectar and pollen rewards, thereby helping them to find rewarding food and assisting pollination by facilitating floral constancy (Giurfa and Sandoz 2012). Exposure to sublethal, field-realistic doses of neonicotinoids has been shown to decrease performance in olfactory conditioning tasks in *A. mellifera* (Decourtye et al. 2003; Decourtye et al. 2004a, 2004b; Han et al. 2010; Yang et al. 2012; Williamson and Wright 2013a; Wright et al. 2015; Piiroinen and Goulson 2016; Iqbal et al. 2018), as well as in the closely related *Apis cerana* (Tan et al. 2015, 2017) and in bumblebees (Stanley et al. 2015). However, others have reported no effect of field-realistic neonicotinoid exposure on rewarded learning in *A. mellifera* (El Hassani et al. 2008, Williamson et al. 2013b) and bumblebees (Piiroinen et al. 2016). Neonicotinoid exposure has also been shown to decrease performance in an aversive conditioning paradigm in honey bees (Zhang and Nieh 2015).

Initial studies suggested that the impact of FPF on *A. mellifera* is relatively low (Glaberman and White 2014). For example, colonies of *A. mellifera* placed next to fields sprayed with FPF showed no reduction in colony strength parameters (numbers of workers, brood, eggs, brood cells, food storage cells, or colony mass; Campbell et al. 2016). More recently, it has been reported that at high acute doses (1.2 a.i. μg per bee) FPF diminishes sucrose responsiveness and impairs performance on associative conditioning tasks (Hesselbach and Scheiner 2018). Significant reduction in performance on olfactory conditioning performance has also been observed in *A. cerana* at field-realistic FPF doses (Tan et al. 2017). In *A. mellifera*, FPF has also been found to interact with nutritional stress to impair thermoregulation and flight dynamics (Tong et al. 2019).

In addition to pesticide exposure, honey bee health is also harmed by disease. *Nosema ceranae* is an emergent microsporidian parasite of *A. mellifera*, causes nosemosis, and is thought to pose a threat to honey bee colony health (Higes et al. 2008, Antúnez et al. 2009)—although the evidence supporting substantial negative impact is sometimes contradictory (Evans and Schwarz 2011, Meixner et al. 2015). Almost nothing is known of the potential impact of this novel microsporidian pathogen on honey bee behavior. However, other pathogens are known to alter learning and memory in *A. mellifera* (Mallon et al. 2003, Iqbal and Mueller 2007). Nosemosis has been associated with impairments of flight behavior (Kralj and Fuchs 2009, Dussaubat et al. 2013), alternations of feeding behavior (Naug and Gibbs 2009), change in pheromone profile (Dussaubat et al. 2013), and acceleration of maturation (Goblirsch et al. 2013, Holt et al. 2013). Thus far, nosemosis alone has been associated with slight impairment of olfactory conditioning (Piiroinen and Goulson 2016) or has had no effect upon *A. mellifera* learning and memory (Charbonneau et al. 2016).

Some evidence suggests that neonicotinoids and *Nosema* interact synergistically to affect honey bee behavior, including impairing a colony’s defense against other pathogens (Alaux et al. 2010) and increasing overall mortality (Retschnig et al. 2014). Indeed, exposure to neonicotinoids during pollination services have been shown to influence susceptibility of *A. mellifera* to *N. ceranae* (Pettis et al. 2013), and exposure of honey bees that were previously infected with *Nosema* to neonicotinoids can significantly increase mortality (Vidau et al. 2011, Pettis et al. 2013). However, Piiroinen and Goulson observed no synergistic interaction between infection with *N. ceranae* and exposure to field-realistic doses of neonicotinoids in *A. mellifera* on learning tasks, despite each factor decreasing performance on its own (Piiroinen and Goulson 2016).

With the potential for a synergistic interaction between FPF and *N. ceranae* in mind, coupled with the relatively unknown impact of both exposure to FPF and nosemosis on learning and behavior in honey bees, we sought to investigate the impact of each of these factors, and their interactions, on the performance of *A. mellifera* under the absolute olfactory conditioning paradigm.

## Methods

We performed three experiments that we describe below, followed by an explanation of the PER process. We also describe how bees were harnessed and how *N. ceranae* spores were quantified. In the first set of experiments, experiment 1a examined the impact of field-realistic FPF while experiment 1b examined the effect of acute LD50 (48 h) FPF. Experiment 2 combined the effects of *N. ceranae* and chronic field-realistic FPF. Following exposure to FPF, we tested the bees under the absolute olfactory conditioning paradigm, using the proboscis extension reflex (PER), during which honey bees extend their proboscises in response to stimulation of the antennae with sucrose solution (Bitterman et al. 1983) to assess acquisition and retention of odor–reward associations.

Bees were collected from July, 2016, to December, 2017 from the University of California San Diego apiary. All colonies were single-box, 10 frame colonies. Colonies were in good health, based upon standard apiary inspection techniques (Dietemann et al. 2013). Upon their initial establishment at the apiary, colonies were treated with fumagilin dicyclohexylammonium (Fumagilin-B) in 25 mg/liter of 2.0 sucrose solution (3.8 liters/colony) to eliminate potential infections and were subsequently not used for 6 mo (Lecocq et al. 2016). Midgut spore counts of control forager bees confirmed that the colonies used were not detectably infected with *N. ceranae*.

Except for experiment 2 (see below), we collected nectar foragers, which exhibit better olfactory learning (Giurfa 2007) by placing feeders containing 1.8 M unscented sucrose at the hive entrance, the same concentration used in our conditioning experiments. Once a bee had begun to drink the sucrose solution, we carefully placed a clear plastic vial over her. Upon satiation, which was assessed as the point at which the bees stopped consuming solution and began to move about the vial, we closed the vial or placed the bees into a communal cage (one per colony; 10–20 bees per cage) for transport back to the laboratory. For each experiment, approximately 10–40 bees were collected each day, usually from two different colonies on a given day.

After returning to the laboratory, we exposed bees to either a field-realistic (approximately 0.44 μg a.i. per bee per day) (experiment 1a and experiment 2) or LD50 (48h) (2.2 μg a.i. per bee) dose of FPF (Sigma Aldrich) (experiment 1b) in 1.8 M sucrose solution and to a 1.8 M control sucrose solution (no FPF). We derived our field-realistic doses and concentrations from Glaberman and White (2014), who reported that apples treated with foliar spray represented a high field-realistic dose at 0.44 μg per bee per day. Concentrations of 4.3 ppm and 4.1 ppm have also been found in the honey stomachs of foragers foraging on rapeseed treated according to the manufacturer’s recommended levels (Glaberman and White 2014). The 48 h LD50 dose was determined, in part, from Glaberman and White (2014), who specified that is 1.2 µg/bee for pure FPF and 3.4 µg/bee for the seed treatment formulation. However, work in our lab suggests that 2.2 μg a.i. per bee of pure FPF is required to produce 50% mortality of bees over 48 h (Tosi, unpublished observations), so we used this higher FPF dose in our experiments.

The experimenters remained blind to the identity of each solution. The FPF and control solutions were prepared only once prior to the beginning of each experiment, were aliquoted into 2 ml Eppendorf tubes, and placed inside plastic storage containers covered in tinfoil to prevent light degradation. The boxes of tubes were stored in a standard freezer (−10°C) and were defrosted at room temperature in light-proof containers prior to use.

### Experiment 1a: 2-d Field-Realistic FPF

The control solution contained plain 1.8 M sucrose, and the experimental solution contained 9.63 mg/liter FPF (0.41 μg a.i. bee/day FPF) in 1.8 M sucrose. Approximately 2 ml of the control or FPF solution was drawn into a syringe, weighed and then carefully introduced into a cage. The syringes were replaced every 24 h (a new 2 ml volume was drawn into a new syringe from the same solution). Bees fed ad libitum, a*s* the entire 2 ml volume was never fully consumed within 24 h. To measure potential evaporation, we drew 2 ml of plain 1.8 M sucrose into a syringe, weighed it, and placed it into an empty cage in the incubator as a control. We recorded evaporation from the control syringe every day. These evaporation controls were run concurrently with the experimental trials. All data were then adjusted to account for this evaporative loss, which was typically less than 1% of syringe mass. We labeled each cage with the treatment and identity of the colony from which its occupants were drawn. Although we often collected bees from two different colonies on any given day, to minimize fighting, we housed the bees in cages according to the colony from which they originated.

We placed the cages into an incubator that was maintained at 36°C temperature and 70% humidity. After 24 h, we removed the cages from the incubators, removed and weighed the syringes, including the control syringe, and placed a new syringe containing the same original solution in each cage. Dead bees were also removed and recorded. This procedure was repeated such that the total duration of FPF exposure was 2 d. Following exposure, we harnessed the bees and performed the PER assay (see below).

### Experiment 1b: Acute LD50 (48 h) FPF

After collecting the bees from the field, they were harnessed (see below), allowed to rest for 1 h in the incubator and were then fed with a pipette 7 µL of premade 2.2 μg a.i. FPF (determined in our lab to be the LD50 dose after 48 h of exposure) in 1.8 M sucrose solution, or the equivalent control (no FPF) solution with a pipette. After feeding, we incubated the bees for another 1 h, after which we tested them with PER.

### Experiment 2: *N. ceranae* and Chronic Field-Realistic FPF

We collected frames with capped brood nearing emergence, which we assessed by uncapping a few cells and checking that the pupal eye colors were purple to black. Once a frame was chosen, we brought it back to the laboratory, and placed it in a sealed nucleus box in an incubator (36°C, 70% humidity) for 24–72 h, until enough bees had emerged to be placed into the experimental conditions.

Once at least 80 individuals had emerged, we collected them from the nucleus box using an aspirator and placed them into individual vials. We placed pipette tips containing 7 µl of either plain 1.8 M sucrose solution, or 40,000 live *N. ceranae* spores freshly harvested from honey bee midguts using standard methods (Dietemann et al. 2013, Fries et al. 2013, BenVau and Nieh 2017) in 1.8 M sucrose solution. The bees remained in the vials until they had consumed all of the solution (up to 8 h) and were then placed into cages. The bees were incubated in their cages for 14 d to allow the *N. ceranae* infections to become established (Forsgren and Fries 2010), and fed 1.8 M sucrose solution during that period. We did not feed bees pollen since pollen can be contaminated with pesticides. Although some studies suggest that feeding young bees sucrose alone may impair their learning (Arien et al. 2015), other learning studies, like ours, do not feed pollen to newly emerged bees (e.g., Tan et al. 2015). Moreover, we observed more than 60% of our control bees exhibiting PER by the final acquisition trials, a level similar to the proportion we observed in experiment 1a and 1b using colony raised foragers that fed on pollen inside their colonies.

Following this 14 d period, we placed half of the bees from each cage into a new cage. Then, following the basic procedure outlined in experiment 1a, we gave half of the cages syringes with either plain 1.8 M sucrose, or 1.8 M sucrose with 9.63 mg/liter FPF. We removed, weighed, and replaced the syringes daily, until 9 d had elapsed. At this point, the bees were harnessed and tested on the PER assay as described in details below.

### Harnessing

**Fig. 1.**
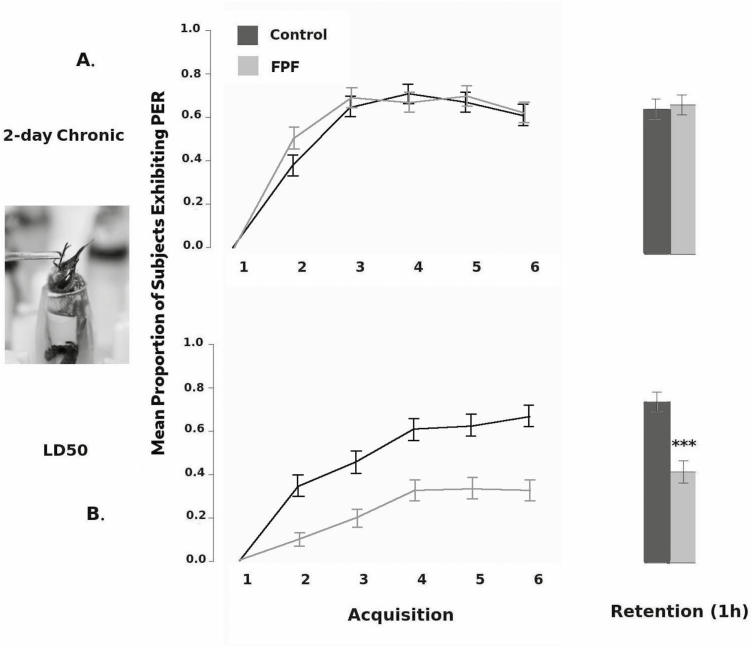
Proportion of PER expressed by controls and FPF-exposed bees during acquisition and retention, tested 1 h after final acquisition trial, for (A) 2-d chronic field-realistic FPF exposure (*n* = 206), and (B) acute LD50(48h) FPF doses (*n* = 186). Error bars are 95% confidence intervals; ANOVA, *** *P* < 0.001. Inset: Bee in the harness apparatus exhibiting a PER when her antennae are stimulated with sucrose solution.

We briefly cold anaesthetized the bees, placing them individually in plastic vials and introducing the vials into a bucket of ice. Once the bee had largely ceased moving, we inserted her head-first into a 1 ml Eppendorf tube with the tip removed. A small 1 cm (approximately) section of a drinking straw with a notch cut out of it was then pushed into the tube behind the bee such that the un-notched side slid under the bee’s wings (Tan et al. 2015). When correctly harnessed, the head of each bee was completely free of the tube, while the bee was prevented from backing out by the straw (see Fig. 1 inset). Each bee received only one assigned treatment and we tested both treatments (control and pesticide) each day.

### Absolute Conditioning of PER in Response to Odor

The collection of newly emerged or forager bees was staggered so that we needed to perform the conditioning assay on only bees from one or two cages at a time. The bees were placed into treatment groups equally for the duration of the experiment so that bees from all treatment combinations (two levels of treatment for experiments 1a and 1b, and four combinations of two levels for each of the two factors for experiment 2) were tested throughout the study. Bees from one cage were harnessed and placed into a tray that contained only bees from that cage. For each group of bees being tested on the same day, we randomly assigned the conditioned odor to be one of two aromatics: geraniol or linalool, common floral odor components that are often used in honey bee olfactory conditioning because bees exhibit good olfactory learning of these compounds (Giurfa 2007). All treatment groups, on any given day, received the same rewarded odor. We exposed the bees to the odor using an aquarium pump (Active Aqua air pump, Hydrofarm Model AAPA25L) and PFTE lined silicon tubing (6 mm inner diameter) attached to a sealed cylinder containing a small piece of filter paper saturated with 10 µl of pure odorant (Smith and Burden 2014). The output of the system was controlled by a solenoid activated by the press of a button. A fan located behind the bee drew the odorant away, and was sucked up a vent via vacuum pump. We wore latex gloves and used clean tweezers to handle the filter paper when inserting it into the cylinder, and the gloves were replaced before each set of acquisition trials.

Every bee in each of the cages experienced six acquisition trials (a full learning sequence). Each acquisition trial consisted of three phases. First, each bee was removed from the tray in harness and placed on a small wooden platform in the apparatus for 3 s (pre-exposure phase). We then exposed the bee to the odor stream for three seconds (conditioned stimulus—CS phase), which was followed by 3 s of odor paired with the presentation of a micropipette dipped in 2.0M sucrose solution to both antennae simultaneously (unconditioned stimulus—UCS phase). For each of the three phases, we noted whether or not the bee unequivocally exhibited a proboscis extension, scoring a 1 if she did, and a 0 for all other responses. After the UCS phase, we returned the bee to the tray. We repeated this procedure for all bees in the tray, and cycled through the entire tray six times. Each tray contained only bees from one treatment group, however all treatment groups were run on the same day, with the order of the treatment groups used randomized between days. The intertrial interval was 10 min. If a bee failed to extend her proboscis during the UCS phase, following stimulus with the sucrose, for two trials in a row, she was removed from the cohort, and her olfactory conditioning data were not included in the analyses.

Following the sixth acquisition trial, we returned the bees in their tray to the incubator for 1 h. At the end of the hour, we performed one retention trial, also consisting of three phases per bee. The pre-exposure and CS phases remained the same as during the acquisition trials, but the UCS phase was replaced by another three seconds of CS that was not paired with sucrose. We expected to see a proboscis extension response (PER) during either the first or second (or both) CS phase of the retention trial if the bees had successfully formed an association between the sucrose solution and the odor stream. Following the retention trial, we euthanized the bees by freezing.

### *Nosema* Spore Quantification

We confirmed *N. ceranae* infection by isolating spores from the dissected midguts of a subset of the bees (five bees per cage) using standard methods (Eiri et al. 2015). We counted spores with a hemocytometer and a compound light microscope (Zeiss Axioskop), recording two independent measures for each sample, and calculating the spore counts as an average of the two counts (Cantwell 1970).

### Statistical Analyses

We performed all analyses using Rstudio v1.0.153 with R v3.4.3 (Venables and Smith 2008). For all experiments, the response variable for the analysis of variances (ANOVAs) for the learning data was always whether or not PER was exhibited during the second of the three trial phases, CS, during which the odor was present but the reward was not. Although binary, this type of data is appropriate for analysis with a traditional ANOVA when the design is balanced, and the error term has at least 40 degrees of freedom (Lunney 1970, Matsumoto et al. 2012). Due to unequal subject attrition as a result of mortality and inappropriate responses, we were left with unbalanced data sets, creating potentially problematic interpretations of the results (Shaw and Mitchell-Olds 1993). To avoid such possibilities, we adopted the conservative approach of balancing our datasets by randomly discarding cases until group sizes were equal (Keppel and Wickens 2004). Specifically, experiment 1a was reduced from 223 to 206 bees, experiment 1b was reduced from 287 to 186 bees, and experiment 2 was reduced from 181 to 160 bees. Experiment 1b was the most unbalanced, due to mortality resulting from the LD50 dose of FPF we administered to the bees. We also analyzed the unbalanced data as they were, which produced identical patterns of results for all of our experiments. We report only the results of the balanced analyses here. For all experiments, we first analyzed the data including odorant (geraniol or linalool) as a covariate. Odorant yielded was not a significant covariate, except for in experiment 1a, for which linalool elicited more PER across all trials than geraniol. Given that the pattern of results for the other factors was unaffected, and in the interest of increasing our statistical power in order to detect weak effects, we did not include odorant in any of the models reported here. Moreover, across all experiments, there was not a single instance of PER occurring during the CS phase of the first acquisition trial with either geraniol or linalool, so there was no evidence that either odor spontaneously elicited PER on its own.

### Experiment 1a

To determine if there was an effect of FPF on the pattern of results across the acquisition trials, we conducted a repeated-measures ANOVA with colony as a covariate, and treatment as fixed between-subjects factor, and trial (excluding the retention trial) as a repeated-measures variable, which is nested within treatment. We also conducted an ANOVA on only the retention trial, with colony as a covariate, and treatment as a fixed factor to see if there were effects on retention of the association between the odor and the reward after 1 h. Additionally, we conducted an ANOVA on the total average volume of sucrose solution consumed by the bees during the 2-d incubation period, with colony as a covariate, and treatment as a fixed factor.

### Experiment 1b

To assess the effect of FPF on the pattern of responses, we used a repeated-measures ANOVA with colony as a covariate, and treatment as a fixed between-subjects factors, and trial as a within-subjects variable. To determine if there was an effect on retention after 1 h, we conducted an ANOVA with colony as a covariate, and treatment as a between-subjects factor.

### Experiment 2

To investigate the effects of exposure to *N. ceranae* and FPF on the pattern of PER during the acquisition trials, we used a repeated-measures ANOVA with colony as a covariate FPF, and *Nosema* as fixed between-subjects factors, and trial as a within-subjects variable. We also ran an ANOVA on the retention trial only, with colony as a covariate, and FPF and *Nosema* as fixed between-subjects factors. Using an ANOVA with colony as a covariate, and, FPF, and *Nosema* as fixed factors, we also assessed the effects of FPF and *N. ceranae* exposure on the total average volume of sucrose solution consumed during the 9-d incubation period corresponding to the time the bees were exposed to FPF as well as *N. ceranae*.

In order to determine the effects of FPF and *N. ceranae* on the survival curves for the four exposure groups during the 9-d incubation period, we ran a Cox Proportional Hazards regression, with *Nosema* and FPF as categorical predictors.

### Dosage, Mortality, and Inappropriate Response Data (all experiments)

To determine effects on mortality and proportion of inappropriate responses (bees that did not respond to the reward on two subsequent acquisition trials), we used Fisher Exact tests, except in the 4×2 case with a sample size greater than 120 (experiment 2), for which we used χ ^2^. When applicable, post hoc tests for Fisher Exact and χ ^2^ were conducted by generating all pairwise comparisons with Bonferroni-corrected Fisher Exact tests. For dosage, mean consumption rates are reported ± 1 standard deviation.

## Results

In our final, balanced analyses, we used a total of 552 foragers from 36 *A. mellifera ligustica* colonies. Of those 36 colonies, three were used in both experiment 1a and experiment 1b, and one colony was used in both experiment 1b and experiment 2.

Performance across the six acquisition trials was analyzed separately from performance on the retention trial, because performance in the acquisition trials indicates how the association was acquired over time, whereas performance in the retention trial indicates how well the association is retained after some period of time has elapsed. For the analyses of acquisition trials, because the performance of each bee was measured repeatedly, trial (acquisition trial, specifically) was a within-subjects effect and treatment (for experiment 2, there were two separate treatment factors) was a between-subjects effect. For the analyses of retention trials, there was no repeated measure, so the only effects analyzed were with respect to between-subjects treatment factors.

### Experiment 1a: 2-d Field-Realistic FPF

The sample sizes for each experiment were the following: 206 bees from nine colonies (July–August, 2016). Over the 2-d exposure, each bee consumed an average of 0.41 ± 0.13 μg a.i. FPF/day, for an average total of 0.83 ± 0.26 μg/bee over both days. There was no main effect of treatment on the overall proportion of PER expressed across all acquisition trials (*F* = 0.2; df = 1, 196; *P* = 0.64), although the colony covariate was statistically significant (*F* = 3.6; df = 8, 196; *P* = 0.0006). There was a statistically significant effect of trial (*F* = 150.9; df = 5, 1020; *P* < 0.0001) on PER, with PER increasing across the subsequent trials, suggesting that bees exhibited learning. The treatment × trial interaction was not statistically significant: there was no difference in the pattern of PER observed across the acquisition trials between the treatment groups (*F* = 0.2; df = 5, 1020; *P* = 0.16; Fig. 1A).

With respect to the retention trial 1 h after the final acquisition trial, the *colony* covariate was statistically significant (*F* = 2.9; df = 8, 196; *P* = 0.0043); however, we detected no significant effect of treatment (*F* = 0.1; df = 1, 196; *P* = 0.85; see Fig. 1A).

In terms of differences in consumption of the solutions, although there was a significant effect of colony (*F* = 13.7; df = 8, 196; *P* < 0.0001) with respect to the total average volume of sucrose solution consumed by the bees, we did not find an effect of treatment on sucrose consumption (*F* = 2.4; df = 1, 196; *P* = 0.12; FPF = 0.098 ± 0.022 g per bee per day; control = 0.094 ± 0.022 g per bee per day).

The mortality rate during the 2-d incubation period did not differ significantly across groups (Fisher Exact, *P* = 0.51). Following the harnessing procedure, significantly fewer FPF bees died than control bees (14.2% vs 8.2%, Fisher Exact, *P* = 0.029), but given the results for experiments 1b and 2, and in other work on FPF in our laboratory, we suspect that this result is due to a Type I error. The number of bees excluded because they failed to respond to the reward during two subsequent acquisition trials did not differ significantly across the treatments (Fisher Exact, *P* = 0.61).

### Experiment 1b: Acute LD50 (48 h) FPF

There were 186 bees from 18 colonies used in this experiment, and data were collected between October, 2016 and December, 2017. At the LD50 (48 h) dose, the colony covariate was significant with respect to the proportion of PER responses produced by bees during the acquisition trials (*F* = 3.6; df = 17, 167; *P* < 0.0001). We also found that there was a significant main effect of treatment (*F* = 26.6; df = 1, 167; *P* < 0.0001), with FPF-exposed bees producing fewer PER responses across all acquisition trials. For the within-bees effects, there was a significant effect of trial (*F* = 71.3; df = 5, 920; *P* < 0.0001). We also detected a significant trial × treatment interaction (*F* = 6.9; df = 5, 920; *P* < 0.0001), indicating that the pattern of PER responses across the acquisition trial differed (see Fig. 1B).

With respect to retention after 1 h, we noted that colony was a significant covariate (*F* = 2.3, df = 17, 167; *P* = 0.005), and that FPF bees exhibited significantly less PER than controls (*F* = 19.5; df = 1, 167; *P* < 0.0001; see Fig. 1B).

Mortality following feeding in the harness differed significantly, with FPF bees experiencing almost twice the mortality rate of controls (41.7% vs 23.9%, Fisher Exact, *P* = 0.0016). Additionally, of the bees that were tested, almost four times as many FPF bees were excluded for failing to respond to the reward on two subsequent trials as control bees (39.2% vs 11.4%, Fisher Exact, *P* < 0.0001).

### Experiment 2: *N. ceranae* and Chronic Field-Realistic FPF

There were 160 bees from six colonies in experiment 2, and data were collected between June and December, 2017. The mean amount of FPF consumed per bee per day in the control + FPF group was 0.29 ± 0.09 μg a.i. per bee per day in contrast to 0.30 ± 0.13 μg a.i. per bee per day in the *Nosema +* FPF group. The total average amount of FPF consumed across the 9-d incubation was 2.57 ± 0.80 μg a.i. per bee for the control + FPF group relative to 2.67 ± 1.20 μg a.i. per bee for the *Nosema +* FPF group.

With respect to the between-subjects treatment groups (every bee was only exposed to one combination of treatment levels), experiment 2 was a 2×2 fully factorial design, allowing us to test for the potential synergy (nonadditive interaction) between exposure to *N. ceranae* and FPF on the performance across all six acquisition trials, and on the single retention trial of the olfactory conditioning task.

**Fig. 2.**
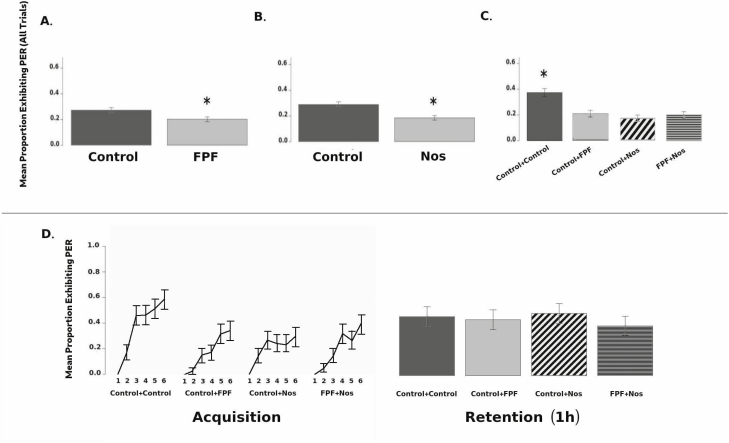
(A) Main effect of 9-d field-realistic exposure to FPF on PER. (B) Main effect of *N. ceranea* infection on PER. (C) Interaction between FPF and *N. ceranae* on PER. (D) Acquisition and retention for all four groups. Total *n* = 160; error bars are 95% confidence intervals; ANOVA, * *P* < 0.05.

Colony was a statistically significant covariate with respect to the proportion of PER observed across acquisition trials (*F* = 10.2; df = 5, 121; *P* < 0.0001). There was a significant main effect of FPF, and bees that were exposed to FPF produced 17% fewer PERs across all acquisition trials (*F* = 6.7; df = 1, 151; *P* = 0.01; Fig. 2A). Bees that were exposed to *Nosema* produced 19% fewer PERs than controls (*F* = 4.4; df = 1, 151; *P* = 0.04; Fig. 2B). Additionally, we detected a significant FPF × *Nosema* interaction (*F* = 6.3; df = 1, 151; *P* = 0.01), with bees who were exposed to both factors producing 18% fewer PERs across trials than controls. Using a Tukey HSD post-hoc test, we determined that it was the control-control group that differed significantly from the *Nosema*-only, FPF-only, and *Nosema +* FPF groups (*P* < 0.001 in all cases), but that the latter three groups did not differ from one another (*P* > 0.05 in all cases; see Fig. 2C). The within-bees analysis revealed a significant effect of trial (*F* = 37.7; df = 5, 780; *P* < 0.0001). Neither the trial × FPF interaction (*F* = 1.8; df = 5, 780; *P* = 0.11), nor the *Nosema* × trial interaction were significant (*F* = 2.081; df = 5, 780; *P* = 0.07), indicating that the pattern of results were similar across these groups. We did detect a significant FPF × *Nosema* × trial interaction (*F* = 2.2; df = 5, 780; *P* = 0.04; see Fig. 2D), suggesting that the pattern of acquisition differed for this group.

Although colony was a significant covariate with respect to the proportion of PER observed during the retention trial (*F* = 5.6; df = 5, 151; *P* < 0.0001), we detected neither a main effect of FPF, nor a main effect of *Nosema* on performance during the retention trial (*F* = 0.4; df = 1, 151; *P* = 0.54, and *F*_1,151_ = 0.1, *P* = 0.72, respectively). The FPF × *Nosema* interaction was also not significant (*F* = 0.5; df = 1, 151; *P* > 0.49; see Fig. 2D).

We failed to detect either an effect of FPF or a FPF × *Nosema* interaction on the total average volume of sucrose solution consumed by the bees (*F* = 0.02; df = 1, 151; *P* = 0.90 and *F* = 1.0; df = 1, 151; *P* = 0.31 respectively; control = 0.029 ± 0.0016 g per bee per day; FPF*-*only = 0.030 ± 0.0062 g per bee per day; *Nosema*-only = 0.028 ± 0.0049 g per bee per day; FPF + *Nosema* = 0.028 ± 0.0042 g per bee per day). Colony was a significant covariate (*F* = 30.3; df = 5, 151; *P* < 0.0001). Interestingly, we found that bees that were infected with *Nosema* consumed less sucrose solution during the incubation period than bees that were not infected (*F* = 36.2; df = 1, 151; *P* < 0.0001; *Nosema* = 0.029 ± 0.0045 g per bee per day; control = 0.028 ± 0.0045 g per bee per day).

**Fig. 3.**
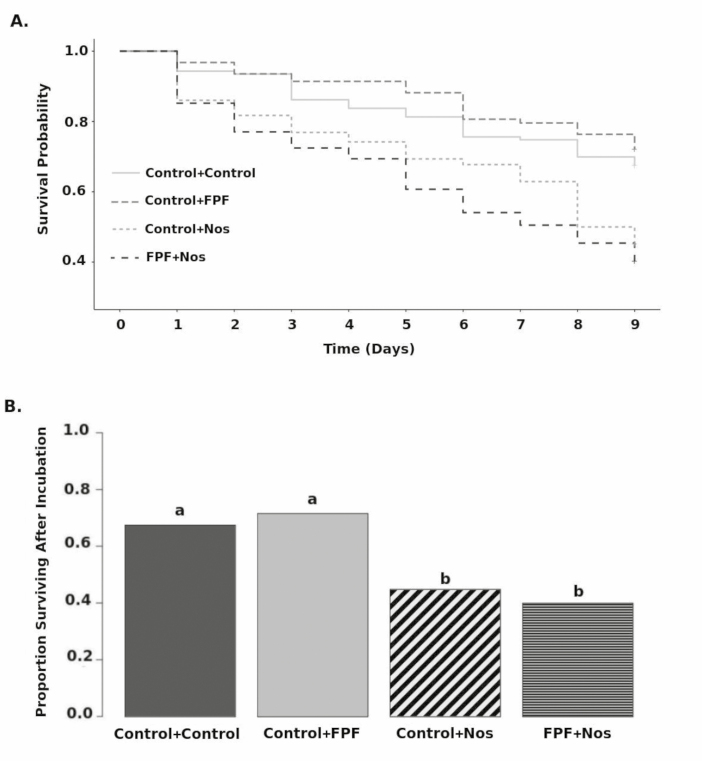
(A) Survival curves by group during 9-d incubation period. Initial *n* = 123 (Control + Control), 93 (Control + FPF), 186 (Control + Nos), and 196 (FPF + Nos) (Cox Proportional Hazard Regression). (B) Proportion of subjects surviving by group after 9-d incubation period. Different letters indicate significant difference (χ ^2^, *P* < 0.05).

With respect to the survival curves during the incubation period, we detected a significant effect of *Nosema* (Wald *χ*^*2*^ = 6.009, *P* < 0.0001; see Fig. 3A), with individuals infected with *N. ceranae* having twice as high of probability of mortality on any given day (4% vs 8%). There was no effect of FPF on survival (Wald *χ*^*2*^  *=* 0.7*, P* = 0.46).

Mortality during the incubation period was significantly different among the groups (*χ*^*2*^ = 41; df = 3; *P* < 0.0001; see Fig. 3B). Bonferroni-corrected pairwise Fisher Exact tests showed that the control + control and control + FPF bees experienced significantly lower mortality than both the control *+ Nosema* and *Nosema* + FPF groups (*P* < 0.001); however, control + FPF did not differ significantly from control + control (*P* = 0.551), and *Nosema +* FPF did not differ significantly from control *+ Nosema* (*P* = 0.353).

Neither mortality following harnessing, nor the proportion of bees failing to respond to the sucrose reward on two subsequent acquisition trials (after which they were excluded) differed significantly among groups (*χ*^*2*^ = 1.9; df = 3; *P* = 0.59, and *χ*^*2*^ = 3.8; df = 3; *P* = 0.29, respectively).

## Discussion

Under chronic, 2-d field-realistic exposure to FPF, we did not find any significant effect of FPF on either acquisition or retention of an olfactory association in *A. mellifera* workers. This short-term field-realistic exposure to FPF also did not significantly alter the amount of sucrose solution consumed by the bees or affect mortality during incubation or the proportion of bees producing abnormal responses during testing. Although significantly fewer FPF-exposed individuals in our experiment died following the harnessing procedure, the actual numerical difference was small, and this result is not consistent with the remainder of our findings. We speculate that this arose because of Type I error and therefore represented a false positive.

Not surprisingly, acute exposure to LD50 (48 h) doses of FPF significantly altered both the acquisition and retention phases of the olfactory conditioning trials. Generally, we detected an overall lower rate of acquisition. That is, a lower percentage PER during the odor-only phase across the FPF-exposed bees. Additionally, mortality following feeding and the proportion of bees excluded due to abnormal responses during testing were both significantly higher in the FPF group. Although such high doses should not be encountered by honey bees, even those foraging in agriculturally intensive areas, the LD50 experiment served as an important positive control, providing expected results from a very high FPF exposure.

We did not detect a synergistic effect of exposure to *N. ceranae* in conjunction with long-term field-realistic exposure to FPF with respect to performance on the olfactory conditioning assay. We did, however, see a reduced degree of acquisition during the acquisition trials for bees that were exposed to *N. ceranae*, FPF, or both. The fact that the groups exposed to either factor individually, or both factors combined, had similarly reduced degrees of acquisition suggests that the effect is a general threshold effect, with exposure to either factor being sufficient to suppress the ability to form the association, or to produce the PER behavior. There was no synergistic effect of *Nosema* and FPF. Interestingly, we did not see a difference in performance on the retention trials across the groups, suggesting that the difference observed during acquisition may not reflect the degree to which the association was formed, but perhaps simply the performance of PER. With respect to mortality, only exposure to *N. ceranae* significantly reduced survival during the incubation period.

Bees infected with *N. ceranae* also consumed significantly less sucrose solution during the incubation period, but exposure to FPF did not change sucrose consumption. Naug and Gibbs (2009) reported that nosemosis increased PER in honey bees; however, they did not look at overall sucrose consumption, but only responsiveness to its presence. Therefore, although one might predict that increased responsiveness should lead to increased consumption, this is not necessarily true. Additionally, Hesselbach and Scheiner (2018) found decreased PER in foragers exposed to FPF. However, their experimental methods were different (acute dose instead of our chronic feeding), and they only saw significant effects at the considerably higher dose of 2.1 μg a.i. per bee. In addition, they measured responsiveness but not consumption. Finally, we did not detect any differences in mortality following harnessing or in the number of bees exhibiting abnormal responses during testing across the groups.

We noted what seemed to be an overall difference in the mean proportion of PER, which reflects the level of expression of the association between the odorant and the reward, observed across the experiments. Several possibilities exist that might explain the differences, including season when the bees were collected, individual differences among the experimenters performing the experiments, and time spent in the incubator in an impoverished nutritional and social environment, or Type I Error. Although our current data cannot be used to distinguish definitively among one or a combination of these alternatives, of the possibilities, time spent in the incubator appeared most closely associated with reduced PER—and in fact both nutrition (Keller et al. 2005, Brodschneider and Crailsheim 2010) and social conditions (Huang et al. 1998, Maleszka et al. 2009) have been shown to play significant roles in brain development and function, and consequently, behavior.

For example, the mean proportion of PER observed across all trials in the control groups for experiments 1a and 1b was 0.53 and 0.49, respectively, but for the control + control group in experiment 2, whose members spent their entire adult lives in the incubator, it was only 0.39. In experiment 1a, the FPF bees exhibited a mean proportional PER of 0.55, whereas the (much higher LD50 dose) FPF-exposed bees in experiment 1b exhibited only 0.24 mean proportional PER across all trials. The mean PER expressed across trials for the control + *Nosema*, control + FPF, and *Nosema +* FPF groups of experiment 3 were 0.21, 0.25, and 0.23, respectively. Taken together, these values suggest that 1) there exists some minimum responsiveness level, around 0.2, that 2) the total dose of FPF matters, hinting that FPF may remain active in the body for some time, and that 3) environmental factors influence performance on absolute olfactory conditioning assays.

Our findings differed from Tan et al. (2017), who saw significant impairments of olfactory conditioning of *A. cerana* when exposed to the same daily dose we used in *A. mellifera*. With the exception of our different chronic exposure periods (2 and 4 d, as opposed to 3 in Tan et al.) our methods did not differ substantially from Tan et al. (2017). However, they reported significantly increased mortality at this same dose, whereas we saw no increase in mortality under either the 2 or 4 d exposure paradigm. This suggests that *A. cerana* may be more sensitive to flupyradifurone than *A. mellifera.* After the 9 d exposure period in experiment 2, we did see some effects during the acquisition trials of the olfactory learning task, suggesting that, perhaps, it takes time for FPF to build up in the body such that it produces behavioral effects. It is also important to note that, whereas neonicotinoids have been found to have similar effects in *A. mellifera* and *A. cerana* (Arena and Sgolastra 2014), the effects of nAChR agonists on different target species are generally difficult to predict a priori (Moffat et al. 2016).

## Data Availability

All data pertaining to this manuscript are located at https://doi.org/10.5281/zenodo.4048983
